# The conservative behavior of dissolved organic carbon in surface waters of the southern Chukchi Sea, Arctic Ocean, during early summer

**DOI:** 10.1038/srep34123

**Published:** 2016-09-23

**Authors:** Kazuki Tanaka, Nobuyuki Takesue, Jun Nishioka, Yoshiko Kondo, Atsushi Ooki, Kenshi Kuma, Toru Hirawake, Youhei Yamashita

**Affiliations:** 1 Graduate School of Environmental Science, Hokkaido University, Sapporo, Japan; 2 Pan-Okhotsk Research Center, Institute of Low Temperature Science, Hokkaido University, Sapporo, Japan; 3 National Institute of Polar Research, Tokyo, Japan; 4 Graduate School of Fisheries and Environmental Sciences, Nagasaki University, Nagasaki, Japan; 5 Faculty of Fisheries Sciences, Hokkaido University, Hakodate, Japan; 6 Faculty of Environmental Earth Science, Hokkaido University, Sapporo, Japan

## Abstract

The spatial distribution of dissolved organic carbon (DOC) concentrations and the optical properties of dissolved organic matter (DOM) determined by ultraviolet-visible absorbance and fluorescence spectroscopy were measured in surface waters of the southern Chukchi Sea, western Arctic Ocean, during the early summer of 2013. Neither the DOC concentration nor the optical parameters of the DOM correlated with salinity. Principal component analysis using the DOM optical parameters clearly separated the DOM sources. A significant linear relationship was evident between the DOC and the principal component score for specific water masses, indicating that a high DOC level was related to a terrigenous source, whereas a low DOC level was related to a marine source. Relationships between the DOC and the principal component scores of the surface waters of the southern Chukchi Sea implied that the major factor controlling the distribution of DOC concentrations was the mixing of plural water masses rather than local production and degradation.

The Arctic Ocean receives approximately 11% of the global discharge despite its upper 200 m layer, constituting only approximately 0.1% of the global ocean volume^1^. Dissolved organic carbon (DOC) concentrations in Arctic rivers are high, particularly during high water-discharge periods, and thus, river runoff adds large amounts of terrigenous dissolved organic matter (DOM) to the upper Arctic Ocean^2^,^3^. In addition to riverine inputs, Atlantic water, Pacific water, autochthonous production, sediments, and sea-ice are potentially important DOM sources in the upper Arctic Ocean^4^. The roles of DOM in marine ecosystems, e.g., as the substrate for microbes and as the factor controlling ultraviolet-visible light penetration, depend on the DOM concentration and composition, which in turn are strongly related to its sources and biogeochemical degradation/alteration^5^. Therefore, it is crucial to evaluate the major factors controlling the DOM distribution of the upper Arctic Ocean.

Terrigenous DOM is widely distributed in the surface of the Arctic Ocean, particularly in shelf regions^1^. There remains considerable controversy about the degradability or conservativeness of terrigenous DOM. The removal of terrigenous DOC, as well as chromophoric DOM (CDOM) in the East Siberian Arctic Shelf and the Hudson Bay, has been noted by its non-conservative behavior against salinity or δ^18^O as tracers of freshwater inputs^6^,^7^. Although some studies have reported a half-life of terrigenous DOC of < 3 years and < 7 years for the Siberian Shelf and the Beaufort Gyre, respectively^8^,^9^, carbon-normalized yields of lignin phenols have indicated that 14–24% of the DOC throughout the upper Arctic Ocean is of terrestrial origin^10^, implying various reactivities of terrigenous DOM. Granskog *et al*.^7^ noted the non-conservative and conservative behavior of riverine CDOM in the Arctic Ocean based on its absorption spectra and flux estimates, respectively^11^.

The melting of sea ice produces another freshwater source in the upper Arctic Ocean. Mathis *et al*.^12^ demonstrated a clear bifurcated relationship between DOC and salinity during the summer in the Chukchi Sea, indicative of the contribution of low DOC sea-ice melt water and high DOC riverine water^12^. The contributions of autochthonous DOC and dissolved organic nitrogen (DON) to the surface water of the western Arctic Ocean have been evaluated based on the spatial distributions combined with salinity and δ^18^O^13^,^14^. The contribution of autochthonous DOM in the upper Arctic Ocean has also been described based on the temporal or spatial distributions of biochemical compounds^15^,^16^,^17^.

Previous studies have successively clarified that several sources, autochthonous production, and removal are possibly important for controlling the DOM distribution in the Arctic Ocean, as mentioned above. These findings were often based on temporal changes in the DOM and/or the spatial distributions of DOM and salinity (and δ^18^O) with a two or three end-member analysis. However, temporal or two/three end-member analyses may not be sufficient to evaluate the major factor controlling the DOM distribution along the margins of the Arctic Ocean where plural water masses, i.e., at least two freshwater sources and several seawater sources, possibly contribute^4^,^7^. Therefore, a novel approach that is independent of physical parameters for water mass identification (i.e., temperature, salinity, and δ^18^O) is necessary to evaluate the major factor controlling the DOM distribution in the regions where a number of seawater sources are evident.

In this study, we determined the spatial distribution of the DOC concentrations and DOM optical properties in surface waters of the southern Chukchi Sea during the early summer of 2013. Several water masses can be discerned in the southern Chukchi Sea, but the DOC concentrations in specific water masses have not been well documented previously^4^,^18^. The water flow through the Bering Strait is northward during most of the year^19^ and is an important source of heat, freshwater, and nutrients into the Arctic Ocean^20^,^21^,^22^,^23^,^24^. The Alaskan Coastal Water (ACW), the Bering Shelf Water (BSW), and the Anadyr Water (AW) enter from the Bering Sea through the Bering Strait, with the ACW on the east, the BSW in the middle, and the AW on the west^23^,^24^. The Siberian Coastal Current occasionally flows southward through the Bering Strait, primarily in the fall and winter^25^. Based on the relationship between the DOC concentration and DOM quality determined by optical analyses, the conservative behavior of the DOC was found to be the major factor controlling the DOC distribution in the southern Chukchi Sea during the early summer of 2013.

## Results and Discussion

### Water masses in the southern Chukchi Sea

**Figure 1:**
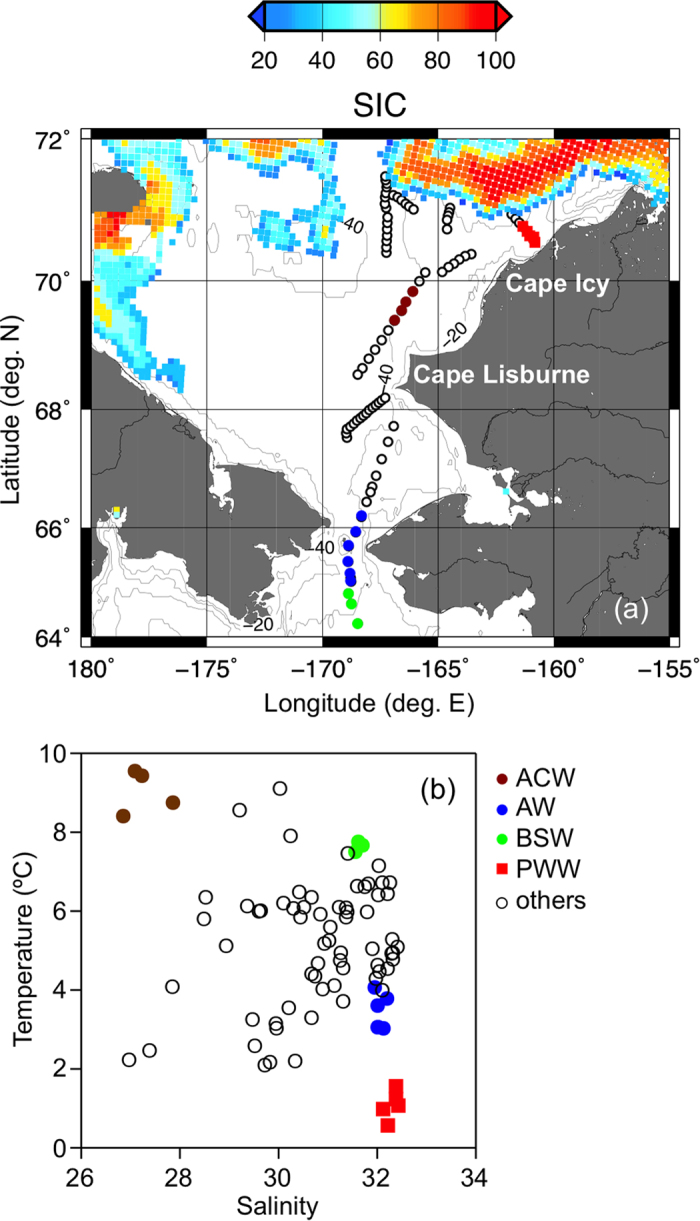
(**a**) Map of the study area showing the sampling locations, the sea-ice distribution during observation, and the location of the specific water masses. Sea ice concentration (SIC, %) on July 12, 2013, estimated by AMSR-2/GCOM-W, is plotted with the color bar. The map was created using The Generic Mapping Tools (version 5.1.1, http://gmt.soest.hawaii.edu). (**b**) Property-property plot between temperature and salinity.

In the early summer of 2013, the warmer, less saline, lower nutrient concentration ACW^23^,^24^, also known as the Eastern Chukchi Summer Water (ECSW)^20^, was distributed to the north of Cape Lisburne (Table 1; Supplementary Fig. S1; Fig. 1). The ACW is known to flow along the Alaskan coast and is affected by Alaskan rivers^23^,^24^. The AW and the BSW, which can be characterized as saline and having higher nutrient concentration compared to the ACW^23^,^24^, were distributed in the vicinity of the Bering Strait (Table 1; Supplementary Fig. S1; Fig. 1). The AW was distinguished from the BSW by temperature and salinity (Table 1; Supplementary Fig. S1; Fig. 1) because the AW can be characterized as cooler and more saline compared to the BSW^23^,^24^. It has been noted that a gradual interface between the AW and the BSW promotes the formation of a combined water mass^23^, and the AW and the BSW have been defined as one water mass, called the Western Chukchi Summer Water (WCSW)^20^; thus, the AW and the BSW identified in this study possibly affected each other.

Sea-ice melting water is another source of freshwater for the upper water column of the southern Chukchi Sea, particularly at the sea-ice edge regions^23^. The other water mass distributed in the Chukchi Sea during summer is the Pacific Winter Water (PWW)^26^,^27^. The PWW is formed during sea-ice formation and thus is characterized as extremely cold, dense, and high in nutrients due to the supply from sediments. Large negative N* in the PWW indicates the influence of sedimentary denitrification^28^. In the present study, the PWW was distributed to the northeast of Cape Icy (Table 1; Supplementary Fig. S1; Fig. 1). The temperature and salinity of the PWW defined in this study were higher and lower than those of the previously defined PWW, respectively^26^,^27^, indicating that this water mass was a “modified” PWW, with mixing of the upwelled PWW and the surface water in the region. Other water masses (others), which were not identified from physicochemical parameters (i.e., temperature, salinity, and nutrient conditions), distributed across the southern Chukchi Sea during the early summer, 2013 (Fig. 1), could be considered as a mixture of specific water masses (i.e., ACW, AW, BSW, PWW, and sea-ice melt water).

### Distributions of DOC concentrations and DOM optical parameters in surface waters of the southern Chukchi Sea during early summer, 2013

**Figure 2:**
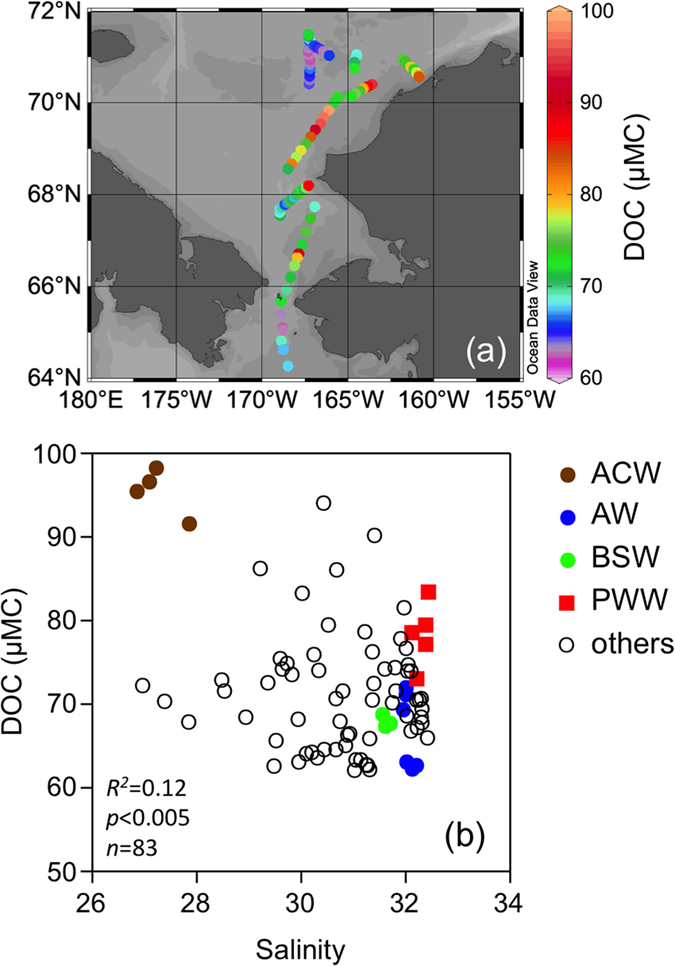
(**a**) Spatial distribution of the DOC concentrations in the surface waters of the southern Chukchi Sea during the early summer, 2013. The map was created using Ocean Data View (version 4.5.3, https://odv.awi.de). (**b**) Property-property plot between DOC concentration and salinity.

The DOC concentration ranged from 62 to 98 μMC in the study area and was similar to, or slightly lower than, values previously observed in the Bering Strait^12^,^18^ and the Bering Shelf 29. The distributional pattern of the DOC was not uniform, but it had a spatial variability related to the distribution of specific water masses (Fig. 2a). The highest DOC concentration was found in the ACW, and the lowest DOC concentration was found in the AW and the BSW, near the Bering Strait, and in the central Chukchi Sea, near the ice edge (Fig. 2; Table 1). The lowest DOC concentration was found in sea ice, even though the range was relatively large between the two samples (Table 1). Similar to the DOC-salinity relationship found for the saline waters of the Chukchi Sea^18^, the DOC concentration did not linearly correlate with salinity (Fig. 2b) or temperature (*R*^*2*^ = 0.15, *p* < 0.001, *n* = 83).

Distribution patterns of optical properties of the DOM (DOM quality) were also not uniform. The spectral slope coefficient between 275 nm and 295 nm (*S*_275–295_; Table 1; Supplementary Fig. S2)^30^, a tracer of terrigenous DOC^1^, had a similar range, as previously observed in the Chukchi Sea^1^ and the Bering Shelf 29. The range of *S*_275–295_ observed in this study indicated a less variable and a small contribution of terrigenous DOC in the southern Chukchi Sea compared to the Eurasian margins of the Arctic Ocean^1^. The values of specific UV absorbance (SUVA_254_; Table 1; Supplementary Fig. S2), an index for the aromaticity of DOM^31^, were lower than those found in the Yukon River basin^32^. Two and one fluorescent components, identified by excitation-emission matrix fluorescence (EEM) combined with parallel factor analysis (PARAFAC), could be categorized as humic-like components (C1 and C2) and a protein-like component (C3), respectively (Supplementary Fig. S3), based on a comparison with previous studies of the Arctic Ocean^33^,^34^,^35^ and the eastern Bering Sea^29^. The distributions of the relative abundance of humic-like C1 (%C1) and protein-like C3 (%C3) were similar to those of the SUVA_254_ and *S*_275–295_, respectively (Supplementary Fig. S2). Similar to the DOC concentrations, the optical parameters were different among the specific water masses (Table 1). The ACW was characterized as having the lowest *S*_275–295_ and %C3 and the highest SUVA_254_ and %C1, whereas the highest *S*_275–295_ and %C3 and the lowest SUVA_254_ and %C1 were evident in the AW. The optical characteristics of the DOM in the PWW and BSW were in the middle ranges and close to those of the ACW and AW, respectively. The SUVA_254_ values in both sea-ice samples were extremely low compared to those in surface waters, and the %C1 and %C3 of the sea ice were lower and higher compared to the surface waters, respectively (Table 1), indicating low aromaticity but richness of the protein-like component in the sea-ice DOM.

**Figure 3:**
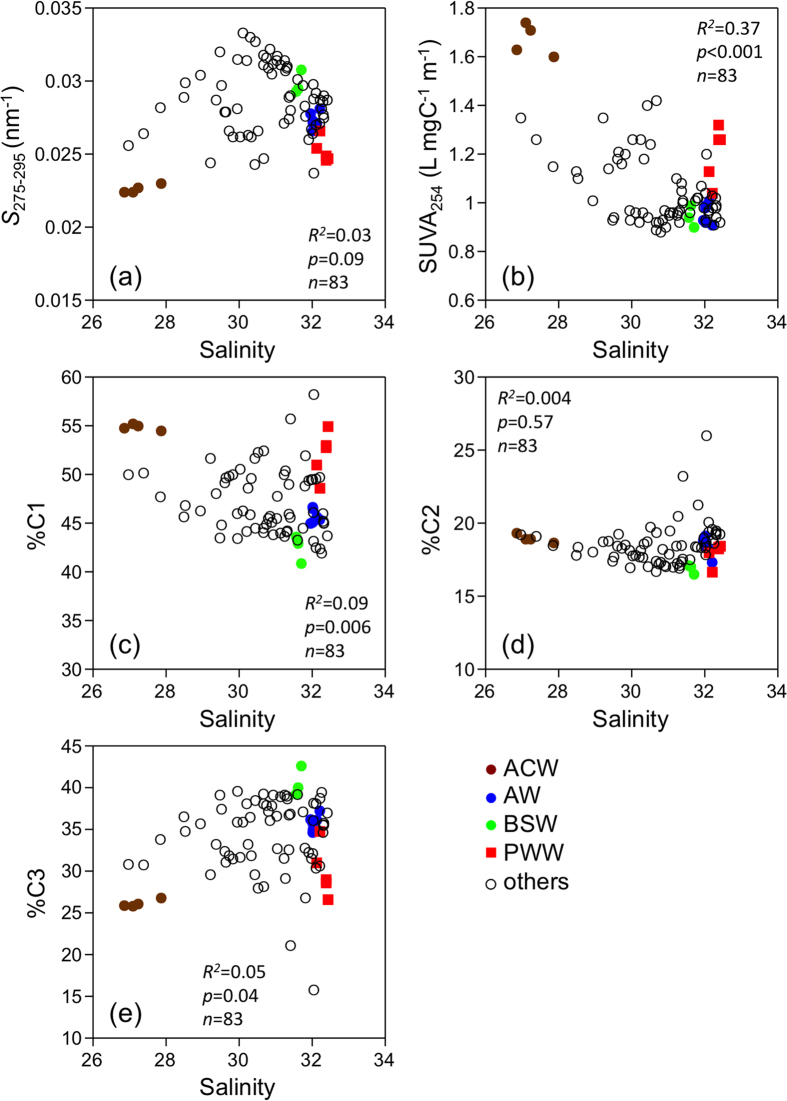
Property-property plots between salinity and (**a**) *S*_275–295_, (**b**) SUVA_254_, (**c**) % C1, (**d**) %C2, and (**e**) %C3.

**Figure 4:**
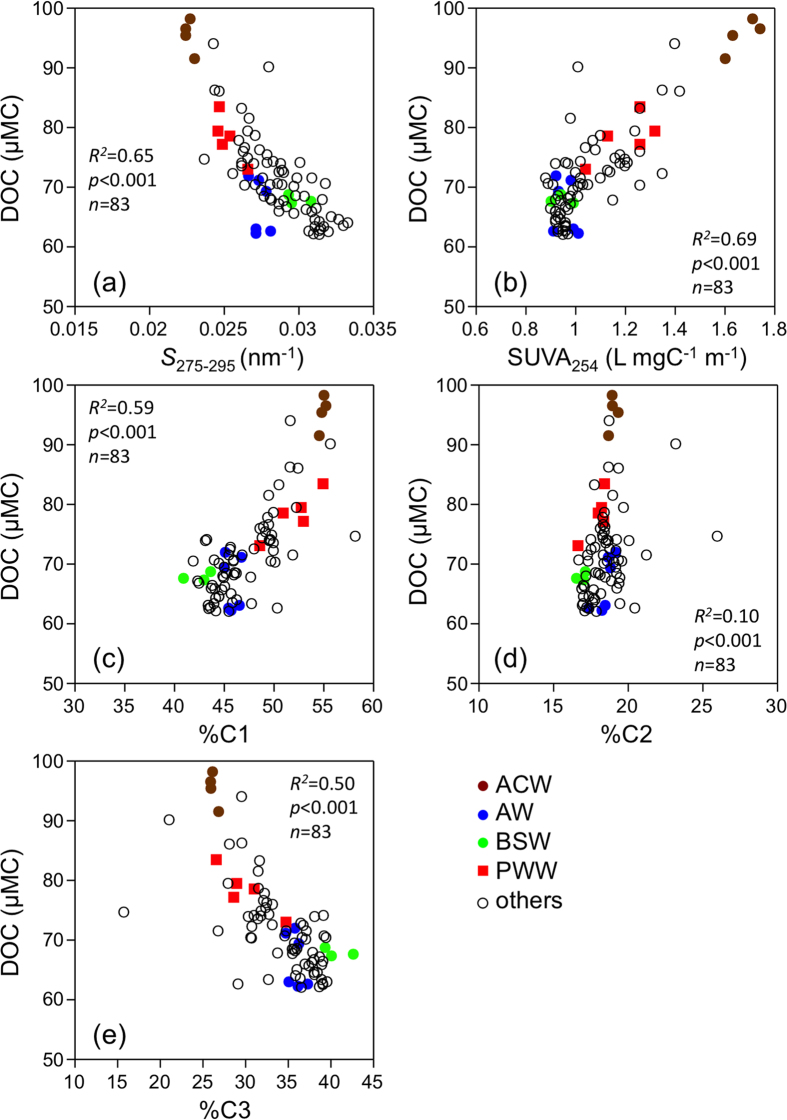
Property-property plots between DOC concentration and (**a**) *S*_275–295_, (**b**) SUVA_254_, (**c**) %C1, (**d**) %C2, and (**e**) %C3.

Even though the SUVA_254_ was weakly correlated with salinity, other optical parameters were not correlated with salinity (Fig. 3), implying that salinity cannot be used to evaluate the conservative nature of the DOM quantity and quality in the Chukchi Sea, as previously observed for the Arctic margins^7^,^9^,^13^. The optical parameters were also not correlated with temperature (*R*^*2*^ < 0.02, *p* > 0.05, *n* = 83 for *S*_275–295_, %C1, %C2, and %C3; *R*^*2*^ = 0.08, *p* = 0.009, *n* = 83 for SUVA_254_). In contrast, significant correlations were evident between the DOC concentrations and DOM optical parameters, except for %C2 (Fig. 4). Such correlations imply that the major factors controlling the DOC concentration and optical properties of the DOM (DOM quality) were similar in the surface waters of the southern Chukchi Sea during the early summer.

### Dynamics of DOC in the surface waters of the southern Chukchi Sea during the early summer, 2013

**Figure 5:**
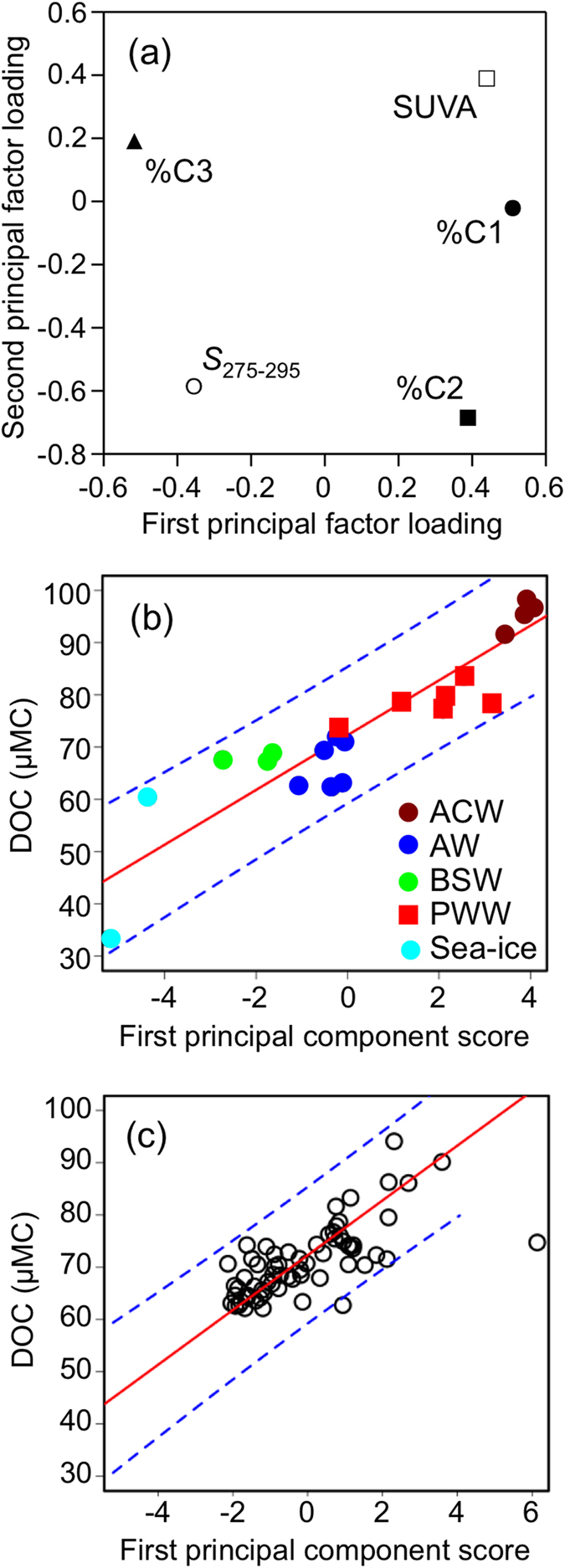
(**a**) Property-property plot between the first and second principal factor loadings of PCA. (**b**) Relationships between the DOC concentration and first principal component score of specific water masses. The solid red and dashed blue lines are the regression line and the 95% prediction intervals of the relationship, respectively. (**c**) Property-property plot between the DOC concentration and first principal component score of the other water masses. The solid red and dashed blue lines are the regression line and the 95% prediction intervals of the relationship derived from specific water masses (Fig. 5b), respectively.

Principal component analysis (PCA) was conducted using optical parameters (i.e., *S*_275–295_, SUVA_254_, %C1, %C2, and %C3) of all surface water and sea ice samples to assess the comprehensive DOM quality in terms of optical properties. The first and second principal components explained 71% and 15% of the variability, respectively. Figure 5a shows the property-property plot between the first and second factor loadings. SUVA_254_, %C1, and %C2 concurrently showed positive first factor loading. *S*_275–295_ and %C3 showed negative first factor loading. The terrigenous DOM was categorized by low *S*_275–295_^1^, high SUVA_254_^3^, and the dominance of humic-like fluorophores^34^,^36^, whereas the marine DOM was characterized by the opposite trends. Thus, the first principal component represented the DOM sources; namely, a positive value indicated a terrigenous origin, whereas a negative value implied a marine origin. Figure 5b shows the relationship between the DOC concentration and the first principal component score for specific water masses. The relationship had differences in the DOM quantity and quality among the Pacific originated waters. The ACW was characterized by a high DOC concentration with terrigenous characteristics, whereas the AW and BSW had low DOC concentrations with marine features. This result implied that the terrigenous DOM from Alaskan rivers, e.g., the Yukon and Kuskokwim Rivers, contributed to the ACW, as indicated in previous studies^26^,^29^, whereas the contribution of terrigenous DOM from Siberian rivers, e.g., the Anadyr River, to the AW was possibly less important. The PWW was characterized by a higher DOC concentration and a greater contribution of terrigenous DOM compared to the AW and the BSW, implying that the DOM with a terrigenous feature may be derived from sediments during sea-ice formation^34^,^37^. The DOM in sea ice was characterized by the lowest DOC concentrations, with marine characteristics.

Interestingly, there was a significant linear relationship between the DOC concentration and the first principal component score of the specific water masses, including sea ice (*R*^*2*^ = 0.85, *p* < 0.001, *n* = 20; Fig. 5b). The relationship showed that high levels of DOC were related to terrigenous characteristics, whereas low levels of DOC were related to marine characteristics. Furthermore, this observation implies that the major factor controlling the distributions of the DOC concentration and DOM quality in other water masses that could not be identified from the physicochemical parameters (Fig. 1) can be evaluated from the linear relationship. Figure 5c shows the scatter plot of the DOC concentration and first principal component score of the other water masses. The regression line and the 95% prediction intervals of the regression obtained from specific water masses (Fig. 5b) are also shown in Fig. 5c. Almost all the data points of the other water masses were distributed between the 95% prediction intervals, indicating that a major factor controlling the distribution of the DOC concentration and DOM quality was the mixing of specific water masses, namely, the ACW, AW, BSW, PWW, and sea-ice melt water, in the surface waters of the southern Chukchi Sea during the early summer of 2013.

Previous studies have suggested that the photochemical process bleaches and alters the optical properties of DOM in the upper waters of the Arctic Ocean^7^,^11^,^34^,^38^,^39^, whereas others have noted that photobleaching of DOM is minor due to the specific conditions of the Arctic Ocean, namely, ice cover, low sun angle, and the strong attenuation of UV radiation by particles and CDOM^3^,^40^,^41^,^42^. The conservative behavior of DOC and DOM quality (Fig. 5b,c) implied that photobleaching of DOM was minor for the surface waters of the southern Chukchi Sea during the early summer. However, it should be noted that the photobleaching of DOM possibly exhibits seasonality with the retreat of sea-ice^29^.

The contribution of autochthonous biomolecules (amino acids, amino sugars, and carbohydrates) to the DOM in surface waters of the western Arctic Ocean during the late summer (late July-August) has been previously described^15^,^17^,^43^. In the present study, EEM-PARAFAC identified a protein-like component (Supplementary Fig. S3); however, the linear relationship between the DOC and the first principal component score was clear (Fig. 5b,c). This result suggested that local production and/or consumption of the protein-like component did not substantially affect the DOC distribution, even though active cycling (i.e., production and consumption) of labile DOM possibly occurred locally. Because the relative abundance of the protein-like component in the AW and BSW was greater than those in the ACW and the PWW (Table 1), the semi-labile protein-like component produced in the Bering Sea was possibly conservatively distributed in the southern Chukchi Sea during the early summer of 2013. The contribution of microbial CDOM has also been described in the western Arctic Ocean^34^,^39^,^44^. It has been well documented that the microbial processing of DOM generates new compounds into the environment^45^,^46^, and part of the CDOM could be showing humic-like fluorescent characteristics and be refractory, such that it can stay in the water for a long time and contribute to carbon sequestration in the ocean^47^,^48^. Such microbial processes should occur in the Arctic Ocean but might be slow due to the low temperature. Even if the production rate of the refractory DOM is low, it accumulates over time, thus contributing to the DOC concentration in the Arctic Ocean as a marine end-member.

The novel approach used in this study clarified that conservative mixing, rather than local production and degradation, was an important factor controlling the DOM distribution in the surface waters of the Chukchi Sea during the early summer, 2013. Levels of humic-like fluorophore can be determined by *in situ* sensors^34^,^49^,^50^. The conservative behavior of a humic-like component implies that the humic-like fluorescence intensity, determined by *in situ* sensors, could be used as one of the physicochemical parameters to determine the water mass, which in turn affects the biological production in the Chukchi Sea^50^. The degradability of terrigenous DOM in the Arctic Ocean is controversial, and the non-conservative distribution of DOC has been noted in the Arctic Ocean, even along the river-influenced margin^6^,^8^,^9^. Such a discrepancy in the dynamics of DOC can be explained by (1) seasonality and/or differences in the shelf characteristics, including the terrigenous DOM compositions, salinity ranges, and time scales of the water mass mixing, or (2) different approaches to evaluate the DOC behavior. It was also noted that the photodegradation of terrigenous DOM mainly occurs beyond the shelf, where the residence time of the water is much longer^44^. The application of the approach used in this study to evaluate the conservativeness of DOM in other shelf regions/basins and other seasons could be helpful to clarify the dynamics of DOM in the Arctic Ocean.

## Methods

Field samples and measurements were collected during the T/S *Oshoro-Maru* C255 cruise, conducted from the southern Chukchi Sea to the Bering Strait in the early summer (July 9–20, 2013) (Fig. 1a). In 2013, the sea ice retreated from the Bering Strait to the north during June, but covered the adjacent to the northernmost stations in mid-July (Fig. 1a). Eighty-three surface water (1–3 m depth) samples were collected using a towed fish metal-free sampling system^51^,^52^. Surface water continuously flowed from the top of the towed fish to the onboard laboratory through a Teflon tube by an air-driven Teflon pump (model PFD-2, Asti co.). The temperature was monitored with a conductivity–temperature sensor (SBE 45, SeaBird ltd.). The time of the water flow from the towed fish to the sensor was less than one minute. Samples for salinity and nutrient analyses were collected without filtration. Samples for the DOC and DOM optical property analyses were filtered through an acid-cleaned 0.22-μm filter unit (Millipak-100, Millipore) at the end of the tubing. The filtrate was collected in pre-combusted borosilicate glass vials and stored immediately at −20 °C until analysis, within four months for optical analyses.

Two sea ice blocks (floes) were collected near the ice edge of the southern Chukchi Sea, using a nylon sling mounted on a vinyl coated stainless frame, which was hung from the ship’s crane. A block of sea ice was cut into small pieces using a ceramic knife. A piece of the sea ice was put into an acid-cleaned HDPE bucket and melted in a dark at room temperature. Immediately after melting, the melted sea ice was filtered through an acid-cleaned 0.22 μm filter (Durapore, Millipore) under a gentle vacuum, collected in pre-combusted borosilicate glass vials, and stored immediately at −20 °C until analysis.

The salinity and nutrient concentrations were measured using a salinometer (AUTOSAL8400B, Guildline Instruments) and an autoanalyzer (QuAAtro2-HR, BL-Tec), respectively. N*, a potential tracer of denitrification, was calculated according to ref. 53. The DOC analysis was conducted by high-temperature combustion using a total organic carbon analyzer (TOC-V_CSH_, Shimadzu). The accuracy and consistency of the measured DOC concentrations were checked by analyzing a deep seawater reference standard (CRM program, Dr. Hansell Lab., University of Miami). After the water sample was thawed and reached room temperature, the absorbance of the sample was measured from λ = 200 nm to 800 nm at 0.5 nm intervals using a spectrophotometer (UV-1800, Shimadzu), according to ref. 54. A 5-cm quartz-windowed cell was used for the analysis. The spectral slope coefficient, between 275 nm and 295 nm (*S*_275–295_), was calculated according to ref. 30. A smaller value of *S*_275–295_ indicated a greater contribution of terrigenous DOM and vice versa^1^. The specific UV absorbance (SUVA_254_), an indicator of the DOM aromaticity, was determined by dividing the absorbance measured at 254 nm by the DOC concentration^31^.

Excitation-emission matrix (EEM) fluorescence was measured using a fluorometer (Fluoromax-4, Horiba), according to ref. 55. The inner filter effect was corrected using the absorbance spectrum, according to ref. 56. Fluorescence intensities were corrected for the area under the water Raman peak (excitation = 350 nm), analyzed daily, and were converted to Raman units^57^. Parallel factor analysis (PARAFAC) was performed in MATLAB (Mathworks, Natick, MA) with the DOMFluor toolbox (version 1.7)^58^. The EEMs of the excitation wavelengths from 250 nm to 450 nm and emission wavelengths from 320 nm to 520 nm were used for PARAFAC modeling, and the three-component model was validated by split half validation and random initialization^58^. The relative abundance of each of the three fluorescent components (%Ci, i = 1 to 3) was calculated using the fluorescence intensity of individual components (C1, C2, and C3), i.e., %Ci = Ci / (C1 + C2 + C3) × 100.

Principal component analysis (PCA) was conducted for the DOM quality (*S*_275–295_, SUVA_254_, %C1, %C2, and %C3) using surface water samples and sea-ice samples (*n* = 85). PCA and regression analysis were conducted using R (version 3.2.1).

## Additional Information

**How to cite this article**: Tanaka, K. *et al*. The conservative behavior of dissolved organic carbon in surface waters of the southern Chukchi Sea, Arctic Ocean, during early summer. *Sci. Rep.* **6**, 34123; doi: 10.1038/srep34123 (2016).

## Supplementary Material

Supplementary Information

## Figures and Tables

**Table 1:** Characteristics of specific water masses.

Water mass	n	Temperature	Salinity	NO_3_^−^ + NO_2_^−^	PO_4_^3−^	N*	DOC	*S*_275–295_	SUVA_254_	%C1	%C2	%C3
		(°C)		(μM)	(μM)	(μM)	(μMC)	(nm^−1^)	(L mgC^−1^ m^−1^)	(%)	(%)	(%)
Alaskan coastal water (ACW)	4	9.0 ± 0.5	27.3 ± 0.4	0.0 ± 0.0	0.2 ± 0.0	−0.6 ± 0.3	95 ± 3	0.023 ± 0.000	1.7 ± 0.1	55 ± 0	19 ± 0	26 ± 0
Anadyr water (AW)	6	3.4 ± 0.4	32.1 ± 0.1	3.8 ± 4.1	1.2 ± 0.4	−2.9 ± 0.5	67 ± 5	0.027 ± 0.001	1.0 ± 0.0	46 ± 1	18 ± 1	36 ± 1
Bering shelf water (BSW)	3	7.7 ± 0.1	31.6 ± 0.1	3.4 ± 2.9	0.4 ± 0.3	−0.5 ± 1.4	68 ± 1	0.030 ± 0.001	0.9 ± 0.0	42 ± 1	17 ± 0	41 ± 2
Pacific winter water (PWW)	5	1.1 ± 0.4	32.3 ± 0.1	3.2 ± 2.5	1.1 ± 0.2	−9.7 ± 0.9	78 ± 4	0.025 ± 0.001	1.2 ± 0.1	52 ± 2	18 ± 1	30 ± 3
Sea-ice	2	n.d.	2.2–4.0*	n.d.	n.d.	n.d.	33, 60	0.023, 0.025	0.2, 0.4	31, 36	16, 16	48, 53

n.d. = not determined.

*Salinity was measured for different pieces of sea ice blocks from DOM analyses (n = 10).
