# Supplementary material for: The conservative behavior of dissolved organic carbon in surface waters of the southern Chukchi Sea, Arctic Ocean, during early summer
Source: Sci Rep. 2016 Sep 23;6:34123. doi: 10.1038/srep34123 (PMC5034254; doi:10.1038/srep34123)
Supplement: Supplementary Information [file srep34123-s1.pdf]

Scientific Reports

Supplementary Information for

The conservative behavior of dissolved organic carbon in surface waters of the southern Chukchi Sea, Arctic Ocean, during early summer

Kazuki Tanaka[1], Nobuyuki Takesue[1,2], Jun Nishioka[2], Yoshiko Kondo[3,4], Atsushi Ooki[5], Kenshi Kuma[5], Toru Hirawake[5], Youhei Yamashita[1,6]

[1]Graduate School of Environmental Science, Hokkaido University, Sapporo, Japan
[2]Pan-Okhotsk Research Center, Institute of Low Temperature Science, Hokkaido University, Sapporo, Japan
[3]National Institute of Polar Research, Tokyo, Japan
[4]Graduate School of Fisheries and Environmental Sciences, Nagasaki University, Nagasaki, Japan, [5]Faculty of Fisheries Sciences, Hokkaido University, Hakodate, Japan
[6]Faculty of Environmental Earth Science, Hokkaido University, Sapporo, Japan

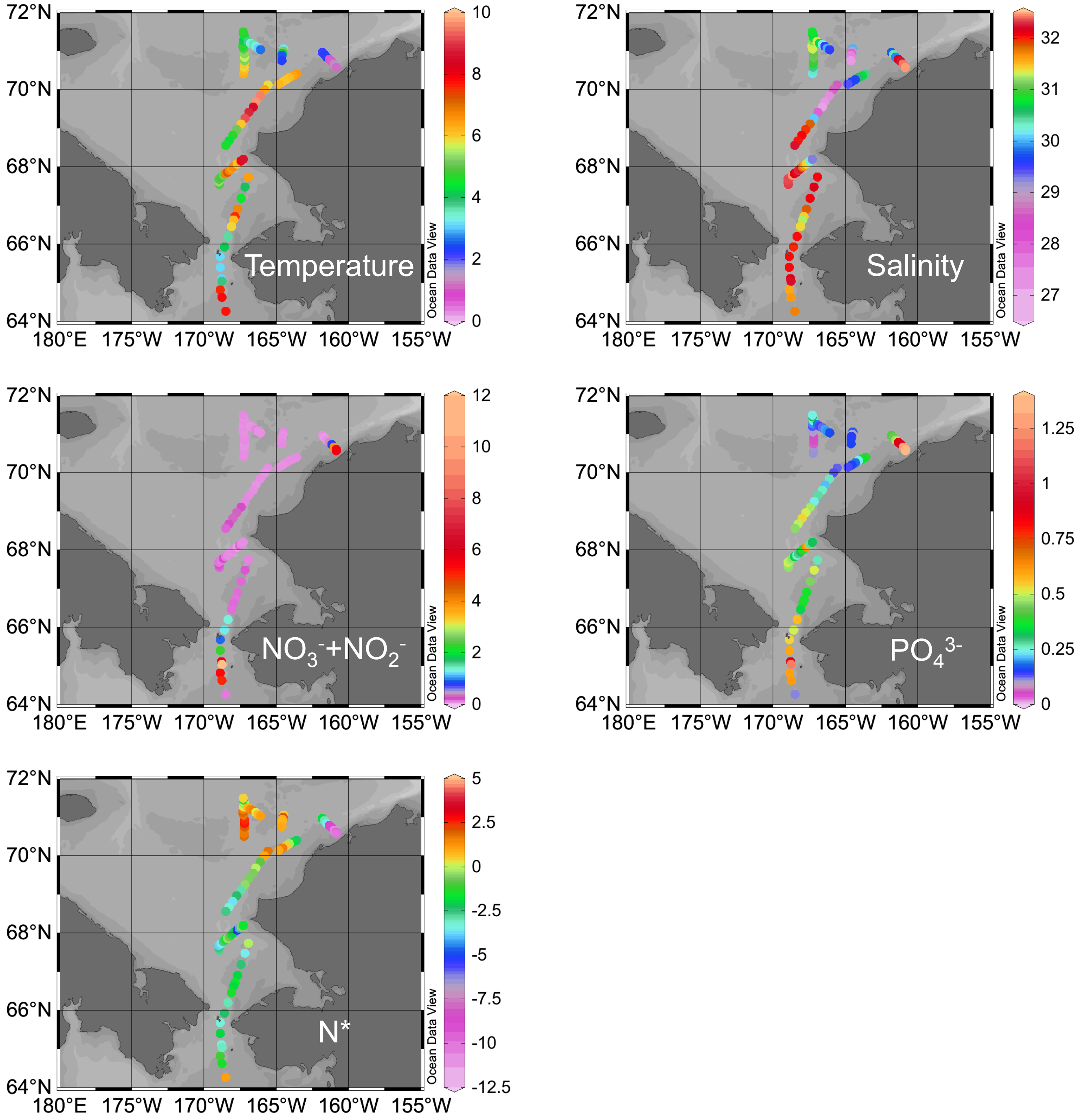

Figure S1. Spatial distribution of temperature, salinity, NO_3^-+NO_2^-, PO_4^{3-}, and N* in surface waters of the southern Chukchi Sea during early summer 2013. Maps were created using Ocean Data View (version 4.5.3, https://odv.awi.de).

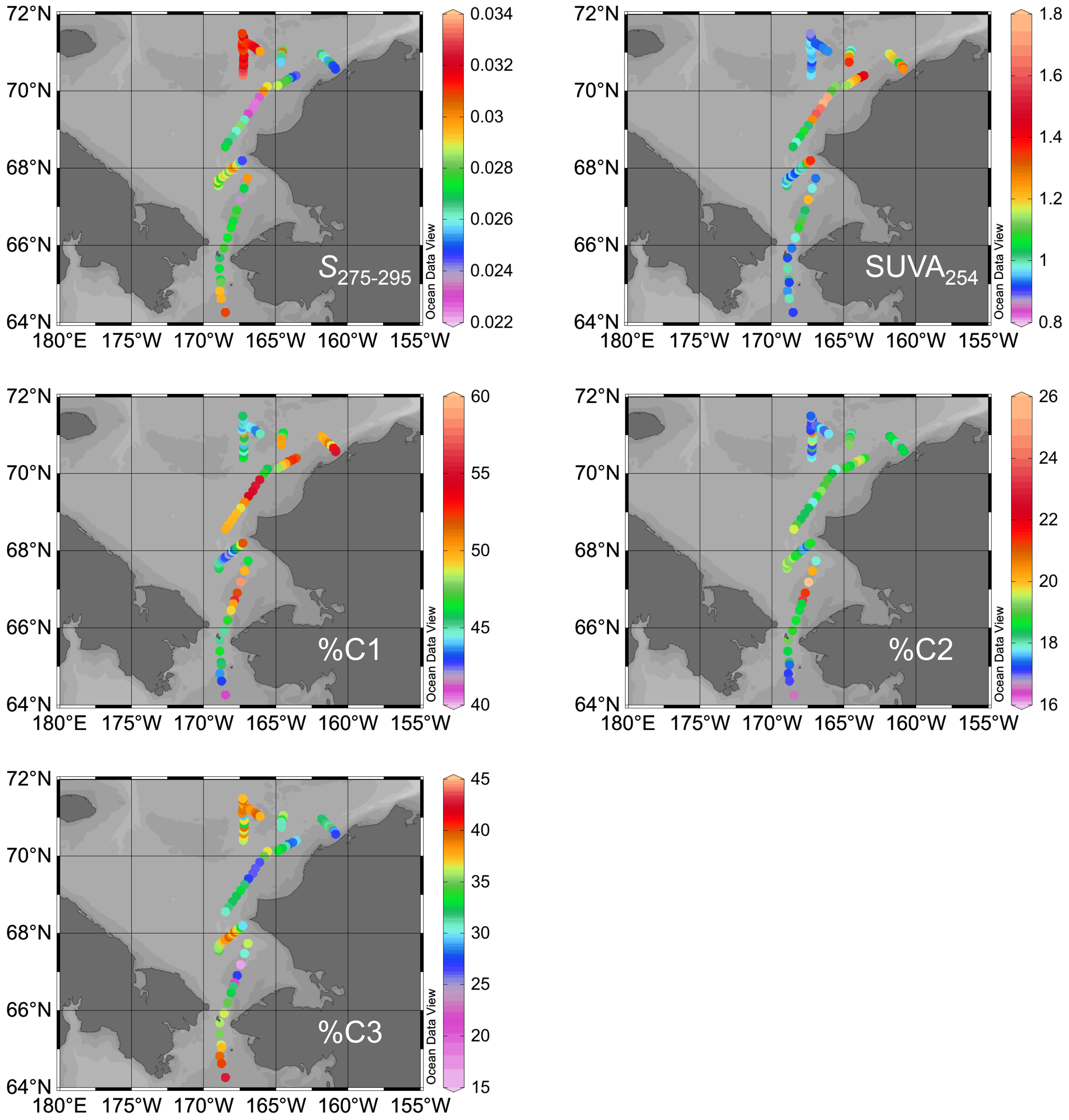

Figure S2. Spatial distribution of $S_{275\text{-}295}$, $SUVA_{254}$, %C1, %C2, and %C3 in surface waters of the southern Chukchi Sea during early summer 2013. Maps were created using Ocean Data View (version 4.5.3, https://odv.awi.de).

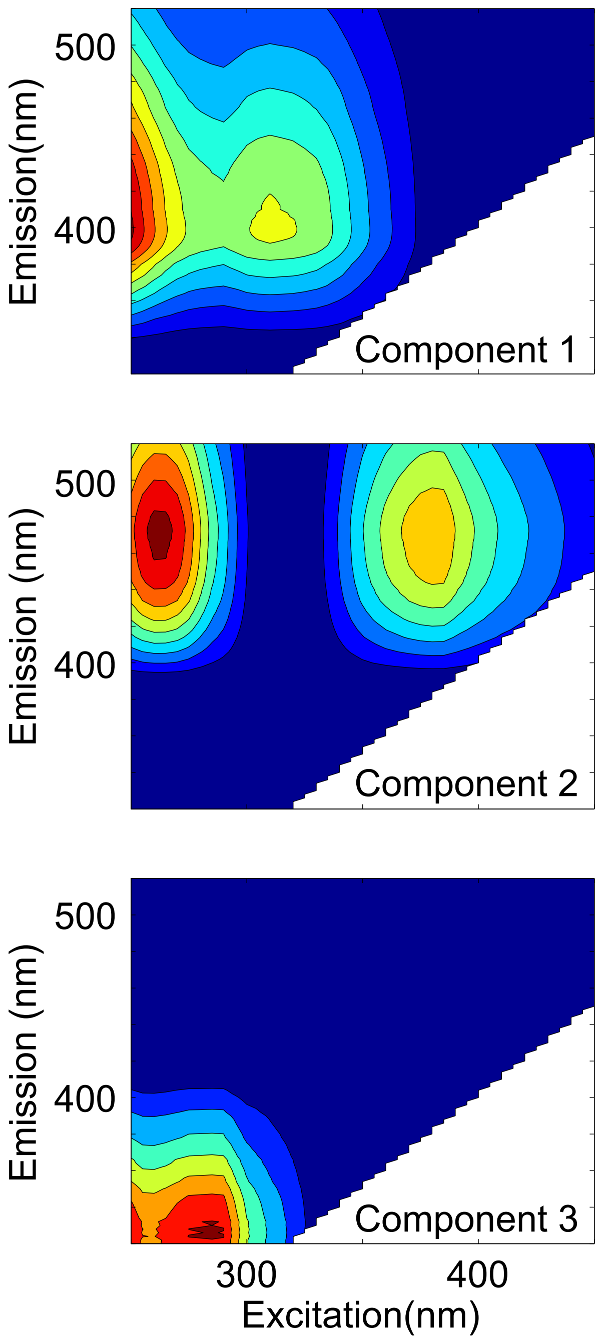

Figure S3. Excitation-emission matrices for PARAFAC components.
